# Appropriate Weaning Practice and Associated Factors among Infants and Young Children in Northwest Ethiopia

**DOI:** 10.1155/2017/9608315

**Published:** 2017-07-20

**Authors:** Liknaw Bewket Zeleke, Mengistu Welday Gebremichael, Yohannes Mehretie Adinew, Kelemeu Abebe Gelaw

**Affiliations:** ^1^College of Health Sciences and Medicine, Wolaita Sodo University, Sodo, Ethiopia; ^2^College of Health Sciences and Medicine, Mekelle University, Mekelle, Ethiopia

## Abstract

**Background:**

The right nutrition from the start of a pregnancy to the child's second birthday has a profound impact on the future health, wellbeing, and success of a child. This can be achieved through proper maternal nutrition during pregnancy, exclusive breastfeeding, and appropriate weaning practice.

**Objective:**

This study was aimed at assessing appropriate weaning practice and associated factors among infants and young children aged 6–23 months in Feres Bet Town, Northwest Ethiopia.

**Methods:**

A community based cross-sectional study was conducted among 351 children aged 6–23 months. Simple random sampling technique was used to select study participants. Interviewer administered questionnaires were used. Bivariate and multivariable logistic regression analyses were employed to identify factors associated with appropriate weaning practice.

**Results:**

Nearly quarter (23.9%) of mothers have practiced appropriate weaning. Proportion of children who started consumption of weaning food timely and met the recommended dietary diversity was 61.5% and 43.9%, respectively. Child age [AOR (Adjusted Odds Ratio): 7.04], husband's occupation [AOR: 6.85], and maternal weaning advice [AOR: 4.38] were positively associated with appropriate weaning practice, while family size [AOR: 0.28] showed negative association.

**Conclusion:**

Appropriate weaning practice was found to be low. Health education at community level and one-on-one advice for mothers in health institutions are highly recommended to improve appropriate weaning.

## 1. Background

Appropriate nutrition during the 1,000-day window period, between the start of a woman's pregnancy and her child's second birthday, is critical to the future health, wellbeing, and success of her child [1]. Because during the first year of life a baby grows more quickly than at any other time and this rapid growth requires proper nutrition [2, 3]. Good nutrition during this period can be achieved through proper maternal nutrition during pregnancy, exclusive breastfeeding (EBF), and appropriate weaning [1].

Malnutrition during the first two years of life results in an irreversible impairments in attaining full potential of physical growth, brain development, and health status of children [2]. Worldwide, more than one-third of all child deaths every year are attributed to malnutrition and it is estimated that 6% of under-five child mortality every year can be reduced through age appropriate infant and young child feeding (IYCF) [3].

World Health Organization (WHO) recommends early initiation of breastfeeding, followed by exclusive breastfeeding for the first 6 months and introducing complementary feeding timely and adequate in amount, frequency, consistency, and variety to address the nutritional needs of the growing infant at 6 months of age with continuing breast feeding up to 2 years [4, 5]. This gradual replacement of milk with solid food as the main source of nutrition is known as weaning or complementary feeding [6]. It is the provision of any nutrient containing foods or liquids other than breast milk [7].

Inappropriate complementary feeding remains among the contributing factors for the persistence of malnutrition in a widespread manner, only quarter of children aged 6–23 months meet the criteria of age appropriate dietary diversity and feeding frequency [8–11]. In Ethiopia, age appropriate infant and young child feeding practice is alarmingly low; as indicated by the national level report of Ethiopian Demographic Health Survey (EDHS) 2011, only 4% of children aged 6–23 months met the criteria of IYCF practices [12]. Thus, the aim of this study was to assess appropriate weaning practice and factors affecting it among mothers having children aged 6–23 months in Feres Bet, Northwest Ethiopia.

## 2. Methods

### 2.1. Study Design, Area, and Period

A community based cross-sectional study was conducted to assess the practice of appropriate weaning among infant and young children aged 6 to 23 months. This study was conducted at Feres Bet Town from March 7 to March 28, 2016. Feres Bet is located at a distance of 374 km from the capital, Addis Ababa, in the northwest direction. A total of 15,342 population lives in the town; of these, 1,199 were under three years old.

### 2.2. Sample Size and Sampling Procedure

The study population was all mothers having children aged 6–23 months in the study area. The sample size was determined by using single population proportion formula with the assumption of 50.4% [13] proportion of minimum meal frequency, 95% confidence level, and 4% margin of error. The sample size was calculated to be 601, but since the total number of mothers having children aged 6–23 months was 852, correction formula was used. Thus, the final sample size was calculated to be 353. Study participants were selected by simple random sampling method using the sampling frame obtained from the town health extension workers. The number of participants selected from each kebele (the smallest administrative unit) was proportional to the number of eligible households. In case of two or more eligible respondents from a single household, lottery method was applied to select one of them.

### 2.3. Data Collection Tools and Procedures

Data were collected by face to face interview technique using a structured and pretested questionnaire adopted with some modification from WHO standardized questionnaire for IYCF practices and EDHS 2011. The questionnaire was first translated to local language Amharic and then back to English to check for internal consistency. Three diploma midwives gathered the data home to home under the supervision of one BSc midwife.

### 2.4. Data Processing and Analysis

The filled questionnaires were checked for completeness and entered into EPI INFO version 3.5.3 statistical software and then exported to SPSS version 20 for further analysis. Descriptive statistics was made. Both bivariate and multivariable logistic regression models were used to identify associated factors. Odds ratios and their 95% confidence intervals were computed and variables with *p* value less than 0.05 were considered as significantly associated with the outcome variable.

### 2.5. Data Quality Assurance

Data quality was controlled by giving training and appropriate supervisions for data collectors. The overall supervision was carried out by the principal investigator. A pretest was conducted on 10% of the questionnaire on one adjacent kebele which was not included in the study. Appropriate modifications were made after analyzing the pretest result before the actual data collection.

### 2.6. Ethical Considerations

Ethical clearance was obtained from the institutional ethical review board of Mekelle University. All study participants were informed about the purpose, benefit, risk, the confidentiality of the information, and the voluntary nature of participation in the study. The interview was conducted in a private environment convenient for the participants. Data were collected only after informed written consent was obtained from each participant that their interview data will be included in publications. Participants found to have mal practices regarding feeding were counseled after data were collected.

### 2.7. Operational Definitions [14]

 
*Weaning* is the process of introducing and making the child accustomed with soft, semisolid, and/or solid foods gradually to replace breast/formula feeding.
 
*Appropriate weaning practice* is the process of introducing soft, semisolid, and/or solid foods by the age of 6 months with age optimal minimum dietary diversity, minimum meal frequency, and continued breast milk feeding.
 
*Minimum dietary diversity* is feeding a child from four and above food groups containing grains, roots, and tubers; legumes and nuts; vitamin-A-rich fruits and vegetables; other fruits and vegetables; dairy products; flesh foods; and eggs.
 
*Minimum meal frequency* is feeding 2 times per day a child aged 6–8 months and 3 times per day for 9–23 months aged child among breastfeeding mothers and 4 times per day among nonbreast feeding mothers.

## 3. Results

### 3.1. Sociodemographic Characteristics of the Study Participants

Out of the expected 353 respondents, 351 mothers along with their infants and young children aged 6 to 23 months were enrolled in the study yielding a response rate of 99.4%. The age of respondents ranged from 15 to 45 years with median age of 25 years. Regarding education, 36.2% of the respondents did not attend formal schooling, while 37.3% of their husbands have attended college or university. All of the participants were from the Amhara ethnic group and orthodox Christian religion. In terms of child characteristics, nearly proportional number of male and female children are involved in the study with a median age of 18.0 months and an interquartile value of 11.0 (Table 1).

### 3.2. Obstetric and Health Care Service Related Characteristics

One hundred sixty-seven (47.6%) of the participants were primipara and only 6.8% had given birth to five or more children. 85.5% had antenatal care (ANC) visits for their last pregnancy and about half (49.7%) of them were advised about child nutrition during attending ANC visit. More than quarter (27.4%) of respondents delivered the indexed child in their home and 22.8% of them had postnatal care (PNC). From total participants, 62.4% received advice on weaning practice from different sources (Table 2).

### 3.3. Weaning Related Findings

Appropriate weaning practice was found to be 23.9% with its composite indicators, timely initiation of weaning food 61.5%, dietary diversified feeding 43.9%, and feeding with the recommended meal frequency per day 69.8%, and 86.6% of children were breastfed (Table 3).

During the period of study, 318 (90.6%) of the participants have started weaning food. Of them, 15.7% started weaning food early, before 6 months of age, 61.5% started in the recommended age range (6–8 months), and the rest 13.4% started lately after 9 months of age. Less than half of the mothers (43.9%) fed their children foods prepared from four/more food groups whereas 15.1% of them provide only one food group. Concerning meal frequency per day, 69.8% of them conformed to the recommended minimum meal frequency, three and more feeding frequency per day. Regarding their breastfeeding status, 13.1% of mothers ceased breast feeding and most (87.2%) of them did it after 12 months.

**Figure 1:**
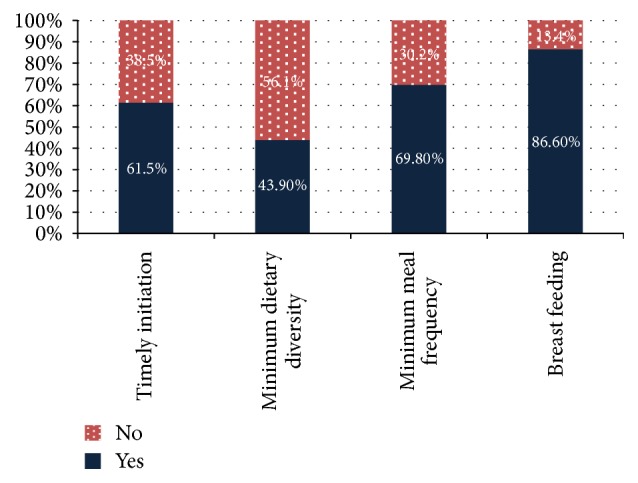
A bar graph on components of weaning practice in Feres Bet, Ethiopia, 2016.

Out of the four indicators used to measure appropriate weaning practice, 43.9% of infants and young children in the study area met minimum dietary diversity feeding, which was found to be the least practiced indicator (Figure 1).

### 3.4. Factors Associated with Weaning Practice

Age of the child, family size, husband's occupation, and mother's exposure to weaning advice were significantly associated with appropriate weaning practice.

Mothers of children aged 9–11 and 12–23 months practiced appropriate weaning 7 times [AOR 7.04; 95% CI (1.578, 31.435)] and 5 times [AOR 5.356; 95% CI (1.58, 18.162)] compared to mothers having 6–8 months aged children, respectively. Mothers whose husbands were governmental employee were about 7 times more likely to practice appropriate weaning [AOR 6.85; 95% CI (2.41, 19.53)] than mothers whose husbands' occupation was self-employed. Mothers who had received advice on weaning practice practiced appropriate weaning about 4 times [AOR 4.38; 95% CI (1.71, 11.29)] compared to those who did not receive. Family size was found to be negatively associated with appropriate weaning practice. Particularly mothers who were living in a family size with four and more members were 71.3% less [AOR 0.28; 95% CI (0.11, 0.73)] likely to practice appropriate weaning compared to mothers living in the family size with three or less. Unexpectedly, this study did not demonstrate association of weaning practice with ANC, place of delivery, PNC visits, and child immunization (Table 4).

## 4. Discussion

The study revealed that appropriate weaning practice was 23.9%. This finding is higher than 2011 EDHS (4%) [12] and studies done in Northern Ethiopia (10.75%) [15] and Ghana (13.8%) [16]. This may be due to the homogeneity of the current study participants who are urban residents. However, the finding is lower as compared to studies done in Indonesia (44.9%) [17] and Zambia (37%) [18]. This might be due to study method difference as the two studies measured appropriate infant and young child feeding mainly based on minimum acceptable diet, which is a combination of meeting minimum dietary diversity, and meal frequency. But the current study measured appropriate weaning from the perspectives of timely initiation, meeting minimum dietary diversity, and meal frequency with continued breast feeding.

It was found that age of index child had significant association with appropriate weaning. Children aged 9–11 and 12–23 months had about seven and five times higher odds of having appropriate weaning compared to children aged 6–11 months. This finding is consistent with studies done in Northern Ethiopia [15], Ghana [16], Tanzania [19], and Nepal [20] in which all studies revealed that older children received age appropriate feeding better than younger children. This might be due to the fact that mothers did not introduce semisolid and soft food to the infants during 6–11 months so that they are fed on animal or canned milk along with breast milk.

Another most important factor significantly associated with appropriate weaning was fathers' occupation. Children whose fathers were government employee were about six times more likely to be appropriately weaned than those whose fathers were self-employed, daily laborers, and students. Studies conducted in different settings demonstrated a significant association between father occupation and age appropriate feeding in different ways. For instance, studies done in Burkina Faso [21] and Indonesia [22] revealed that belonging to agricultural worker and nonworking fathers were a risk factor for not receiving minimum acceptable diet. In contrary to the studies done in Guinea, studies from Niger and Bangladesh revealed that children whose fathers were engaged in agricultural works received better age appropriate feeding than those whose fathers were engaged in nonagricultural work [21, 23]. Mainly those who are government employee are egger to accept scientific findings, advertisements from the media, and recommendations from community health extension workers. This difference may be due to study area and population variation. The current study was conducted on urban area in a limited number of agricultural workers.

Exposure of mothers to weaning advice was found to be significantly associated with appropriate weaning practice. Mothers who received weaning advice tend to practice appropriate weaning four times more likely than those who did not receive. This finding is consistent with a study done in United Kingdom [24]. Though the current study did not show association between health care service utilizations and weaning practice, mothers might receive the information from urban health extension workers who provide service from home to home.

Family size showed a negative association with appropriate weaning practice. Children from a family size, containing four and more members, were 71.3% less likely to get appropriate weaning than those from family size of three and less. This finding is in line with findings of EDHS 2011 [12] and a study done in Pakistan [25], which conclude that the second and third born children had a higher risk of not gaining weaning foods in the recommended age than those of first born infants. This might be related to food affordability problem with increased family size.

## 5. Conclusion

Infant and young children aged between 06 and 23 months receiving appropriate weaning were low compared with other countries. Age of a child, mothers' exposure to weaning advice, and being a child of government employee were positively associated, while family size showed a negative association with appropriate weaning practice. Health education at community level and one-on-one advice for mothers in health institutions are highly recommended to improve appropriate weaning.

## Figures and Tables

**Table 1:** Sociodemographic characteristics of participants in Feres Bet, Amhara, Ethiopia, 2016.

Characteristics	Category	Frequency	Percentage (%)
Age			
Median 25.00, range 8			

Mothers' occupation	Housewife	171	48.6
	self-employed	89	25.4
	Gov't employee	68	19.4
	Others^*∗*^	23	6.6

Family size	≤3	160	45.6
	4–6	164	46.7
	≥7	27	7.7

Husband occupation	Self-employed	200	57
	Gov't employee	138	39.4
	Others^*∗*^	13	3.6

Sex of child	Male	173	49.3
	Female	178	50.7

Age of child	6–8	51	14.5
	9–11	38	10.8
	12–23	262	74.7

^*∗*^Students and daily laborers.

**Table 2:** Obstetric and child characteristics of participants in Feres Bet, Amhara, Ethiopia, 2016.

Characteristics	Categories	Frequency	Percentage (%)
Parity	Primipara	167	47.6
	Multipara	160	45.6
	Grand multipara	24	6.8

ANC visit	Yes	300	85.5
	No	51	14.5

Nutrition advice during ANC	Yes	149	49.7
	No	151	50.3

Place of delivery	Home	96	27.4
	Health center	190	54.3
	Hospital	64	18.3

PNC visit	Yes	80	22.8
	No	271	77.2

Immunization	Yes	305	86.9
	No	46	13.1

Weaning advice	Yes	219	62.4
	No	132	37.6

**Table 3:** Weaning related findings in Feres Bet Town, Amhara, Ethiopia, 2016.

Characteristics	Category	Frequency	Percentage (%)
Introduction of complementary foods	Yes	318	90.6
	No	33	9.4

Age of initiation of complementary foods	Before 4 Months	21	6.6
	4-5 Months	34	10.7
	6–8 months	216	67.9
	9–11 months	22	6.9
	12–23 Months	25	7.9

Reason for Starting complementary foods^*∗*^	Inadequacy of breast milk	112	35.2
	Appropriate age	295	92.8
	Sickness of mother or child	12	3.8
	Inconvenience for work	40	12.6
	Breast Problem	10	3.1

First foods^*∗*^	Liquids	68	21.4
	Semi solid/mashed foods	23	7.2
	Porridge/gruel	244	76.7
	Family food	40	12.6
	Whole Cow milk	54	17.0

Dietary Diversity/day^*∗*^	One	53	16.7
	Two	65	20.4
	Three	46	14.5
	Four and above	154	48.4

Meal Frequency/day	One	9	2.8
	Two	71	22.3
	Three	129	40.6
	Four and above	109	34.3

Breast Feeding Cease	Yes	47	13.4
	No	271	86.6

Age of ceasing breast feeding	Before 11 Months	6	12.8
	From 12–23	41	87.2

^*∗*^Proportion cannot be 100% (it is based on multiple option questions).

**Table 4:** Factors associated with weaning practice in Feres Bet Town, Ethiopia, 2016.

	Appropriate weaning practice		OR (95% CI)	
	Yes	NO	COR [CI]	AOR [CI]
*Maternal education*				
No formal schooling	12	115	1	1
Primary education	9	47	1.83 [0.72, 4.64]	1.701 [0.42, 0.74]
Secondary and above	63	105	5.75 [2.93, 11.25]	1.028 [0.26, 0.99]
*Occupation*				
Housewife	33	138	1	1
Self-employed	6	83	0.30 [0.12, 0.75]	1.24 [0.34, 4.45]
Gov't employee	40	28	5.97 [3.23, 11.04]	2.08 [0.59, 7.376]
Others	5	18	1.16 [0.40, 3.35]	3.64 [0.59, 22.13]
*Family size*				
≤3	45	115	1	1
≥4	39	152	0.65 [0.40, 1.07]	0.28 [0.11, 0.73]^*∗*^
*Husband education*				
No formal schooling	9	78	1	1
Primary education	5	42	1.03 [0.32, 3.27]	0.50 [0.11, 2.17]
Secondary and above	66	76	7.52 [3.50, 16.1]	1.33 [0.33, 5.24]
*Husband occupation*				
Self-employed and others	19	148	1	1
Governmental employee	61	48	9.89 [5.38, 18.20]	6.85 [2.41, 19.53]^*∗*^
*Child age*				
6–8	6	47	1	1
9–11	12	24	3.92 [1.30, 11.72]	7.04 [1.57, 31.43]^*∗*^
12–23	66	196	2.64 [1.07, 6.45]	5.35 [1.58, 18.16]^*∗*^
*Parity*				
Primipara	55	112	1	1
Multipara	29	155	11.62 [6.27, 21.56]	1.33 [0.46, 3.79]
*ANC*				
No	5	44	1	1
Yes	79	223	3.11 [1.19, 8.14]	0.42 [0.10, 1.75]
*Place of Delivery*				
Home	9	87	1	1
Health Institution	75	179	4.05 [1.93, 8.46]	1.61 [0.55, 4.71]
*PNC*				
No	24	56	1	1
Yes	60	211	1.50 [0.863, 2.63]	0.65 [0.28, 1.48]
*Weaning advice*				
No	75	144	1	1
Yes	9	123	7.11 [3.42, 14.80]	4.38 [1.71, 11.29]^*∗*^

NB: ^*∗*^significantly associated, COR: crude odds ratio, and AOR: adjusted odds ratio.

## Abbreviations

ANC: Antenatal care
DHS: Demographic and Health Survey
EDHS: Ethiopian Demographic and Health Survey
IYCF: Infant and young child feeding
PNC: Postnatal care
WHO: World Health Organization.

## Data Access

Data supporting this finding are available upon request.
